# Caregivers’ perspectives on feline chronic kidney disease in Portugal: a questionnaire-based study

**DOI:** 10.1177/1098612X251377486

**Published:** 2025-12-15

**Authors:** Tomás Rodrigues Magalhães, Ana Luísa Lourenço, Ronald Jan Corbee, Inês Guerra, Felisbina Luísa Queiroga

**Affiliations:** 1 Department of Veterinary Sciences, University of Trás-os-Montes and Alto Douro (UTAD), Vila Real, Portugal; 2 Animal and Veterinary Research Centre (CECAV), UTAD, Vila Real, Portugal; 3 Associate Laboratory for Animal and Veterinary Sciences (AL4AnimalS), UTAD, Vila Real, Portugal; 4 Department of Animal Science, UTAD, Vila Real, Portugal; 5 Department of Clinical Sciences, Faculty of Veterinary Medicine, Utrecht University, Utrecht, The Netherlands; 6 Hospital do Gato, Lisbon, Portugal

**Keywords:** Chronic kidney disease, questionnaire-based study, renal disease, veterinary communication, caregiver perspective

## Abstract

**Objectives:**

The study aimed to assess caregivers’ perspectives on feline chronic kidney disease (CKD).

**Methods:**

People living in Portugal caring for a cat with CKD were invited to participate in an online questionnaire.

**Results:**

In total, 405 responses were considered, with most participants (n = 309, 76.3%) dealing with feline CKD for the first time. International Renal Interest Society (IRIS) CKD staging was explained to 260 (64.2%) respondents, but only 188 (46.4%) confirmed that blood pressure was assessed at diagnosis. The recommendation of a renal diet was received by 387 (95.6%) respondents, but only 341 (84.2%) gave it, and, of the latter, 139 (40.8%) were ultimately offering at least some non-renal food daily. Medications, supplements/nutraceuticals and subcutaneous fluid therapy were administered at home by 255 (63.0%), 204 (50.4%) and 205 (50.6%) respondents, respectively. Among the caregivers who used a phosphate binder (n = 123), at least 46 (37.4%) were giving it without food. After diagnosis, the caregiver–cat emotional bond remained the same, improved or worsened in 285 (70.4%), 106 (26.2%) and nine (2.2%) cases, respectively. Most respondents felt completely informed by their veterinarian (n = 331, 81.7%), complied with the recommended monitoring frequency (n = 377, 93.1%) and believed that their experience would not influence future decisions to get a new cat (n = 221, 54.6%). Clinical signs and proteinuria, the need for medication and nutraceuticals/supplements, the administration of erythrocyte-stimulating agents and subcutaneous fluids, and the monitoring frequency were higher (*P* <0.05) in cats at late IRIS CKD stages.

**Conclusions and relevance:**

Most caregivers surveyed felt informed; however, some recommendations were not completely followed. Veterinarian–caregiver communication must be improved to ensure greater adherence to medical recommendations, and an early diagnosis should be pursued to minimise the caregiver burden.

## Introduction

Given the progressive and irreversible nature of feline chronic kidney disease (CKD), the therapeutic goal is to slow its progression, minimise the associated clinical signs, improve quality of life and increase survival.^1,2^ For that purpose, disease management should be tailored to each patient based on currently accepted international guidelines, and considering that each recommended intervention is supported by varying degrees of quality of evidence, according to the benefits described in the literature.^2–4^ Dietary phosphorus restriction, through the introduction of a renal diet, is one of the most evidence-based recommendations, particularly in cats at CKD stages 2–4.^1,5^ In line with this, the administration of phosphate binders has been recommended when serum phosphate levels are not being controlled by the diet alone or when a transition to a low-phosphorus diet has not been successfully accomplished.^2,6^ Another common intervention applied to these patients is intravenous or subcutaneous fluid therapy to address dehydration and electrolyte and acid–base imbalances.^7,8^ Severe and sustained increases in the urine protein:creatinine ratio (UPC) and blood pressure (BP) are currently considered to be indications for the prescription of appropriate medications, such as angiotensin-converting enzyme inhibitors, angiotensin receptor blockers and/or calcium channel blockers.^9,10^ To date, it has not been demonstrated that these medications increase longevity; yet, the evidence supporting their impact on the improvement of quality of life was graded as ‘poor’ for proteinuria management and ‘good’ for hypertension management.^3^ Some additional treatments, namely those to correct anaemia and metabolic acidosis, are generally reserved for more advanced CKD stages (3 and 4), when these complications are more likely to occur and when ensuring quality of life is prioritised over delaying the disease’s progression.^2,11^

Caregivers play a key role in ensuring that the aforementioned treatments are carried out properly, either by being responsible for implementing them at home or by taking their cats to receive further veterinary care.^12–14^ Their commitment to these treatment recommendations, as well as to follow-up appointments for clinical and laboratory monitoring, is crucial because of their clear impact on the disease’s course and prognosis.^3^ For example, an appropriate dietary intervention has been associated with significantly longer survival times, which can be achieved only if the caregiver ensures that the renal diet is properly consumed on a daily basis.^15,16^ Therefore, in order to enhance the care provided, it is important to evaluate the caregivers’ performance and identify opportunities for improvement, which has motivated several studies, although mostly limited to the UK^14,17^ and/or USA.^12,13^ As a result of cultural, socioeconomic and demographic differences, this sort of research should be expanded to other countries.

Clinical management of feline CKD was recently evaluated by the authors in a questionnaire-based study addressed to veterinarians working in Portugal, which identified some critical points that warrant further refinement to maximise cats’ prognosis.^18^ To complete this assessment and provide an overview regarding the current care of these patients in Portugal, the aim of the present study was to evaluate the caregivers’ perspective about several aspects related to their cats’ renal disease.

## Materials and methods

To obtain information from cat caregivers in Portugal, an online questionnaire was designed, consisting mainly of multiple-choice and check-box questions, with a few short-answer inquiries included (see supplementary material). An optional open question was also incorporated at the end of the questionnaire, so the respondents could share additional information about their cat’s condition, as well as comments regarding their own experience. The data obtained from this final question were not subject to formal analysis but rather used to provide the authors with more insights into the cat caregivers’ perception and their common concerns. Before response collection, the questionnaire underwent an external validation process, involving a group of 10 cat caregivers of different ages, education levels and social backgrounds, to ensure that it would be easily understandable by the wider target population.

The final version of the questionnaire received a favourable ethical opinion from the Ethics Committee of the University of Trás-os-Montes and Alto Douro (Doc18-CE-UTAD-2024) and was made available through Microsoft Forms. Respondents were required to be aged over 18 years, live in Portugal and be caring for a cat with CKD at the time of participation. An image with a QR code and a short video were created to be shared on social media, and Portuguese veterinarians were also asked to collaborate by raising awareness among their clients. For the latter, all veterinary clinics and hospitals listed on the Directorate-General for Food and Veterinary Medicine (DGAV) website (https://www.dgav.pt, accessed on 24 January 2024) were contacted via email. In total, the period of active response collection lasted 13 months (January 2024 to February 2025).

All the data obtained were downloaded into an Excel spreadsheet (Excel version 2502; Microsoft Corp). Responses to short-answer questions, as well as those freely submitted when the ‘other’ option was selected, were grouped based on similarities in their content. Entries that failed to meet the inclusion criteria were rejected. The final database was then imported into SPSS Statistics software version 29.0.0.0 (IBM Corp) for descriptive analysis. χ^2^ and Fisher’s exact tests were also applied to investigate associations between the International Renal Interest Society (IRIS) CKD stage and some variables of clinical interest, with *P* <0.05 considered statistically significant.

## Results

**Figure 1:**
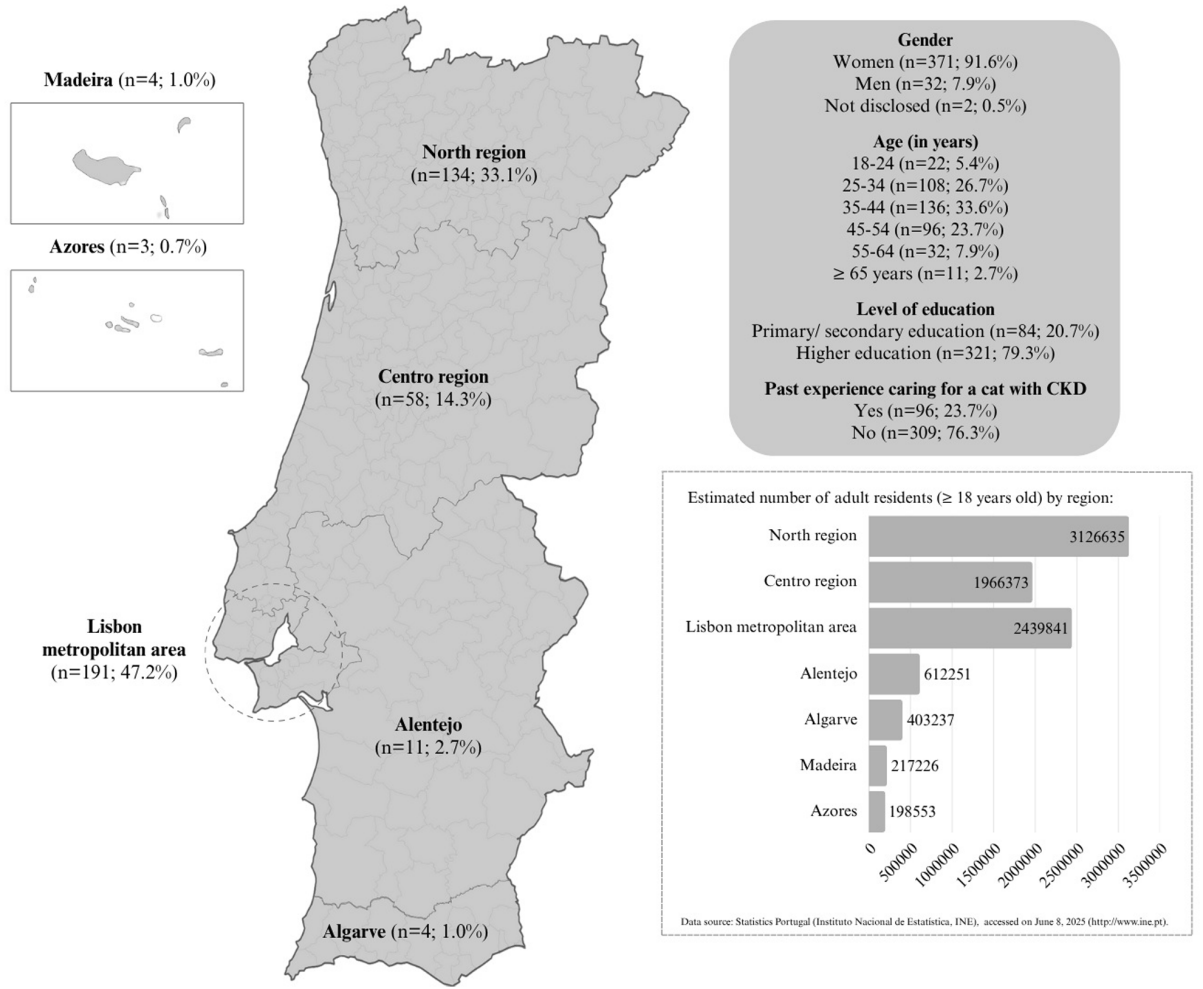
Sociodemographic characterisation of the 405 caregivers surveyed

In total, 451 caregivers participated, but 46 responses were ultimately excluded: 45 referring to cats that had already died at the time of participation and one due to the absence of a diagnosis. Therefore, 405 responses were considered valid and were included. A predominance of women, working-age adults, highly educated individuals and people living in the two largest urban areas (Porto and Lisbon) was found among respondents (Figure 1). As the target population was unknown and the questionnaire was freely accessible to all caregivers who met the criteria for participation, the response rate could not be determined.

### Characteristics and environment of affected cats

Most cats were European (n = 228, 56.3%) or domestic shorthair/longhair (n = 134, 33.1%) and neutered (n = 391, 96.5%). Their median age at diagnosis was 10 years (range 4 months to 21 years). Concomitant diseases had been diagnosed in 175 (43.2%) cats, with periodontal disease (n = 41, 10.1%) and hyperthyroidism (n = 22, 5.4%) being the most common. The environment and resources provided to these cats are described in Table 1.

**Table 1:** Description of the environment and resources provided to the 405 cats

	Frequency
Environment	
Shared with other cats	263 (64.9)
Litter boxes	
More than the total number of cats	110 (27.2)
Distributed across different spaces of the household	119 (29.4)
Water supply	
Water bowls in multiple locations	249 (61.5)
Automatic fountains available	205 (50.6)
Water and food bowls placed next to each other	188 (46.4)
Diet (before diagnosis)	
Commercial dry food	396 (97.8)
Commercial wet food	241 (59.5)
Homemade food	13 (3.2)
Raw food	11 (2.7)

Data are n (%)

### Clinical presentation, diagnosis and staging

At diagnosis, clinical signs were present in 298 (73.6%) cats, particularly weight loss (n = 172, 42.5%), polydipsia (n = 148, 36.5%), loss of appetite (n = 147, 36.3%), vomiting (n = 146, 36.0%), lethargy/weakness (n = 100, 24.7%) and polyuria (n = 94, 23.2%). In contrast, 107 (26.4%) cats showed no evidence of any abnormal clinical manifestations and were diagnosed through routine tests. The diagnosis was reportedly established mostly based on blood tests (n = 394, 97.3%), urinalysis (n = 288, 71.1%) and abdominal ultrasonography (n = 282, 69.6%).

Of the caregivers, 260 (64.2%) recalled having had the IRIS staging system explained, while 75 (18.5%) indicated they had not received that information and 70 (17.3%) were unsure. Of the 260 respondents who were informed, 69 (26.5%), 77 (29.6%), 41 (15.8%) and 15 (5.8%) stated their cat was diagnosed at stage 1, 2, 3 and 4, respectively. Substaging was also investigated, with 318 (78.5%) and 188 (46.4%) of all caregivers reporting that the UPC and BP had been measured at diagnosis, respectively. Among those who confirmed the UPC assessment, 201 (63.2%) and 71 (22.3%) replied that this urinary parameter was abnormal and normal, respectively. Of all cats whose BP was recorded, 95 (50.5%) had a normal value, 47 (25.0%) had hypertensive values and one (0.5%) was reported to be hypotensive. For the rest (n = 45, 23.9%), their caregivers were unable to specify the result.

### Nutritional management

A therapeutic renal diet was recommended to 387 (95.6%) caregivers, mostly by a veterinarian (n = 383). Of all respondents, 345 (85.2%) confirmed that they were aware of the benefits of a renal diet (Table 2). In addition, some misconceptions were mentioned, namely that the renal diet had fat (n = 2) and potassium (n = 2) restriction, a higher phosphate (n = 1) and protein (n = 1) content, or no protein at all (n = 1).

**Table 2:** The benefits of the therapeutic renal diet described by the 405 caregivers surveyed

Benefits reported by caregivers	Frequency
Protein restriction	130 (32.1)
Supports kidney function	84 (20.7)
Controls/slows disease progression	71 (17.5)
Phosphate restriction	57 (14.1)
Sodium intake control	22 (5.4)
Improved urinary tract health	17 (4.2)
Water supply	15 (3.7)
Reduction of clinical signs	13 (3.2)
Better protein quality	12 (3.0)
Increased survival time	10 (2.5)
Improved general condition/quality of life	8 (2.0)
Not aware of its benefits	60 (14.8)

Data are n (%). Only benefits described by five or more respondents were considered

Most caregivers had switched their cat to a renal diet (n = 341, 84.2%). Within this group, most dietary transitions were completed in 2 weeks or less (n = 190, 55.7%) or without a documented gradual protocol (n = 112, 32.8%), differing from the standard recommendations given for these cases. Of those who did transition their cat to a renal diet, 139 (40.8%) respondents were regularly offering other foods (Table 3).

**Table 3:** Food transition period and percentage of renal diet in the cat’s daily feeding, according to 341 caregivers

	Frequency
Food transition period carried out	
No transition/immediately	112 (32.8)
<1 week	107 (31.4)
1–2 weeks	83 (24.3)
3–4 weeks	32 (9.4)
5–6 weeks	1 (0.3)
7–8 weeks	2 (0.6)
>8 weeks	4 (1.2)
Percentage of the renal diet consumed	
100% (total) of the daily feeding	202 (59.2)
<100% but >75% of the daily feeding	69 (20.2)
⩽75% but >50% of the daily feeding	34 (10.0)
⩽50% but >25% of the daily feeding	16 (4.7)
⩽25% but >0% of the daily feeding	8 (2.3)
0% (none) of the daily feeding	12 (3.5)
Type(s) of renal diet provided	
Dry commercial food	307 (90.0)
Wet commercial food	227 (66.6)
Homemade food	13 (3.8)
Raw food	3 (0.9)
Reasons not to offer a renal diet exclusively	
Partial or complete food refusal	93 (27.3)
Difficulty in controlling prescribed food intake (multi-cat household)	14 (4.1)
Cat’s specific preference for a non-renal wet food	14 (4.1)
Cost/financial burden of the renal diet	11 (3.2)
Other nutritional management required (concomitant disease)	9 (2.6)
No noticeable improvement in the cat’s overall condition	2 (0.6)
Other	4 (1.2)

Data are n (%)

### Additional therapeutic approaches

Overall, 255 (63.0%), 204 (50.4%) and 205 (50.6%) cats were receiving medications, nutraceuticals/supplements and subcutaneous fluids, respectively (Table 4). In addition, 33 (8.1%) were on erythrocyte-stimulating agent (ESA) therapy.

**Table 4:** Medications and nutraceuticals/supplements administered and main aspects of the subcutaneous fluid therapy, according to the 405 caregivers surveyed

	Frequency
Medications	
Medication to control proteinuria (eg, ACE inhibitor or ARB)	148 (36.5)
Antiemetic (eg, maropitant)	88 (21.7)
Appetite stimulant (eg, oral or transdermal mirtazapine)	81 (20.0)
Medication to control hypertension (eg, CCB or ARB)	64 (15.8)
Histamine type-2 receptor antagonists (eg, famotidine)	36 (8.9)
Proton pump inhibitors (eg, omeprazole)	21 (5.2)
Thiamazole	6 (1.5)
Gabapentin/pregabalin	5 (1.2)
None	150 (37.0)
Nutraceuticals/supplements	
Phosphate binders	123 (30.4)
Multivitamin concentrates	50 (12.3)
Iron and B vitamin products	30 (7.4)
Omega-3 fatty acid supplements	24 (5.9)
Potassium supplements	17 (4.2)
*Lespedeza*-based products	13 (3.2)
Probiotics	13 (3.2)
Glycosaminoglycans-rich products	6 (1.5)
Tryptophan	5 (1.2)
None	201 (49.6)
Fluid therapy administration	
Applied only at home	120 (29.6)
Applied only at the veterinary clinic/hospital	55 (13.6)
Applied in both locations	30 (7.4)
Not applied	199 (49.1)
Not sure	1 (0.2)
Reasons for not receiving subcutaneous fluids	
Not recommended by the attending veterinarian	162 (40.0)
No need at the time	28 (6.9)
Cat did not tolerate the procedure	15 (3.7)
No willingness to subject the cat to the procedure	6 (1.5)
No noticeable improvement in the cat’s general condition	3 (0.7)

Data are n (%). Only medications and nutraceuticals/supplements reported by five or more respondents were considered

ACE = angiotensin-converting enzyme; ARB = angiotensin receptor blocker; CCB = calcium channel blocker

Of the 201 cats that had proteinuria at diagnosis, half (n = 100) were on antiproteinuric medication at the time the caregiver completed the questionnaire, whereas among the 47 cats that were hypertensive at presentation, 27 (57.4%) were being treated for that condition.

Of the 123 caregivers who administered a phosphate binder, the majority provided it with food, either in a single meal (n = 30, 24.4%) or across two or more meals (n = 32, 26.0%). A subset (n = 46, 37.4%) administered the binder directly into the cat’s mouth, independent of mealtimes. Among the same 123 caregivers, 101 (82.1%) reported having previously attempted a dietary transition, and 64 (52.0%) administered the phosphate binder in addition to an exclusive renal diet.

### Caregiver–cat emotional bond, caregiver–veterinarian communication and disease monitoring

**Figure 2:**
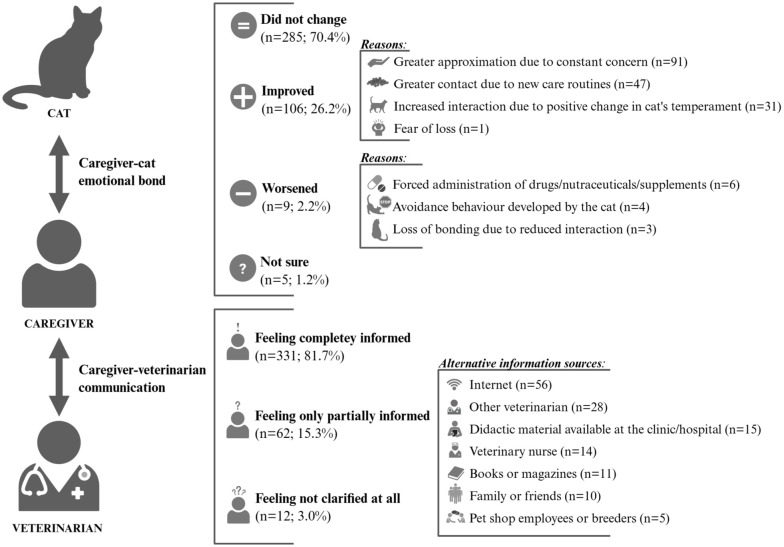
Caregiver–cat emotional bond and caregiver–veterinarian communication after diagnosis, according to the 405 caregivers surveyed (*Created in BioRender. Magalhães, T. (2025) https://BioRender.com/rrltckw)*

For most respondents, the emotional bond with their cat remained the same after diagnosis (n = 285, 70.4%), and the information given by the veterinarian was considered sufficient to feel fully informed (n = 331, 81.7%) (Figure 2).

Moreover, according to the majority (n = 235, 58.0%), when their cat was in a stable stage of the disease, the veterinarian recommended reassessment every 4–5 months or more often, and 377 (93.1%) reported complying with the medical recommendation (Table 5).

**Table 5:** Monitoring frequency recommended by the veterinarian for cats in a stable stage of renal disease, according to the 405 caregivers surveyed

	Frequency
Monitoring frequency recommended by their veterinarian	
More than once a month	22 (5.4)
Monthly	56 (13.8)
Every 2–3 months	126 (31.1)
Every 4–5 months	31 (7.7)
Every 6 months	98 (24.2)
Every 7 or more months	27 (6.7)
Only when the cat became worse	45 (11.1)
Adherence to follow-up recommendation received	
Yes	377 (93.1)
No	28 (6.9)
Reasons for non-adherence	
Cat’s stress during transportation and/or at the clinic/hospital	16 (4.0)
Cost/financial burden of the appointments and/or tests	13 (3.2)
Not thinking the cat needed to be monitored so regularly	6 (1.5)
Lack of time for veterinary visits	6 (1.5)
Forgetting to schedule an appointment	2 (0.5)

Data are n (%)

Most respondents (n = 388, 95.8%) indicated an understanding that feline CKD is a progressive condition managed through supportive care aimed at slowing progression and enhancing quality of life. A smaller proportion (n = 13, 3.2%) expressed the belief that a curative outcome might be possible. In addition, 121 (29.9%) respondents found the information provided about the disease sufficient to understand its general aspects, while 128 (31.6%) believed that more educational resources were needed. Overall, 221 (54.6%) respondents stated that the impact the disease had on their cat’s life and their own would not influence future decisions regarding having a new cat, with 81 (20.0%) believing it would and the remaining 103 (25.4%) not committing to any answer.

### Potential associations between IRIS CKD stage and variables of clinical interest

When the analysis was narrowed to cats with a known IRIS CKD stage at diagnosis (n = 202), some statistically significant associations were identified (Table 6).

**Table 6:** Associations between disease stage (early vs late) and variables of clinical interest in the 202 cats with known International Renal Interest Society CKD stage at diagnosis

	Frequency		
	Early CKD stages (n = 146)	Late CKD stages (n = 56)	*P* value
Clinical signs at presentation			<0.001
Yes	97 (66.4)	55 (98.2)	
No	49 (33.6)	1 (1.8)	
Proteinuria at diagnosis			<0.001
Proteinuric	67 (45.9)	40 (71.4)	
Non-proteinuric	44 (30.1)	3 (5.4)	
Not measured	10 (6.8)	4 (7.1)	
Don’t know/don’t remember	25 (17.1)	9 (16.1)	
Transition to a renal diet			0.378
Yes	127 (87.0)	46 (82.1)	
No	19 (13.0)	10 (17.9)	
Medication administration			<0.001
Yes	73 (50.0)	48 (85.7)	
No	73 (50.0)	8 (14.3)	
Nutraceutical/supplement administration			0.036
Yes	70 (47.9)	34 (60.7)	
No	76 (52.1)	21 (37.5)	
Not specified	0 (0.0)	1 (1.8)	
ESA therapy			0.026
Yes	11 (7.5)	8 (14.3)	
No	133 (91.1)	44 (78.6)	
Don’t know/don’t remember	2 (1.4)	4 (7.1)	
Fluid therapy			<0.001
Yes	56 (38.4)	41 (73.2)	
No	90 (61.6)	15 (26.8)	
Recommended monitoring frequency			0.004
More than once a month	4 (2.7)	9 (16.1)	
Monthly	17 (11.6)	14 (25.0)	
Every 2–3 months	47 (32.2)	12 (21.4)	
Every 4–5 months	13 (8.9)	4 (7.1)	
Every 6 months	50 (34.2)	13 (23.2)	
Every 7 or more months	10 (6.8)	2 (3.6)	
Only when the cat became worse	5 (3.4)	2 (3.6)	

Data are n (%). *P* value represents the level of significance

Other variables were evaluated, such as breed, sex/reproductive status, age (≤ 10 years vs > 10 years) and blood pressure at diagnosis, but were not significantly associated with disease stage

CKD = chronic kidney disease; ESA = erythrocyte-stimulating agent

## Discussion

The present findings showed that most cats already had clinical signs at diagnosis of CKD, with these being more frequent among late-stage patients.^19^ However, over one-quarter of the cats were identified solely through routine tests, which is a higher relative percentage than those reported in other studies.^14,20^ This difference may be caused by a greater awareness in recent years about the importance of check-up screening tests for early diagnosis.^21^ Moreover, the IRIS staging system was explained to a larger percentage of caregivers compared with a previous survey-based study involving UK-based owners (64.2% vs 20.5%),^14^ reflecting that IRIS staging has been increasingly adopted by veterinarians over time. This approach may benefit from further reinforcement to support caregiver understanding of the progressive nature of feline CKD and its associated prognosis. Enhanced communication and education efforts could potentially increase the proportion of caregivers who are fully informed and who recognise the primary goals of CKD management. For substaging, UPC was determined in most cats, but BP measurement was seemingly often overlooked, which is consistent with the very limited percentage of Portuguese veterinarians (19.1%) who reported performing BP measurement on all their newly diagnosed cats.^18^ Some of the reasons behind this include equipment unavailability, difficulty in performing and/or interpreting BP measurement, stress/lack of patient compliance, and caregivers’ financial and/or time constraints. Particular obstacles must be identified to find solutions to systematise this procedure, allowing early detection of systemic hypertension, which is a highly prevalent condition in these patients.^22^ Still, it should be noted that since the diagnosis and staging were reported by the caregiver and were not cross-checked with the attending veterinarian’s records (to maintain full anonymity), there may be some discrepancies due to misunderstanding/miscommunication.

The treatment recommendations published by IRIS^2^ and in the ‘ISFM consensus guidelines on the diagnosis and management of feline chronic kidney disease’^3^ have been the most widely applied in the therapeutic management of feline CKD. Although both were developed by a panel of experts and reflect current evidence, these guidelines should be interpreted as general recommendations rather than strict rules, as each case has its specific circumstances. Either way, dietary intervention is consistently considered the foundation of this approach, often relying on the prescription of a renal diet, which is typically characterised by restricted phosphorus content, moderate to restricted high-quality protein, elevated concentrations of omega-3 fatty acids, and added potassium and B vitamins.^1,5^ Beyond adequate nutritional supply tailored to the patient’s needs, it is crucial that the diet ensures the required calorie intake, as cats with significant weight loss tend to have poorer outcomes.^23,24^ According to the findings of the present study, a renal diet was recommended by the veterinarian for most cats, but in 20.5% of those who transitioned, it represented 75% or less of their daily feeding, which is in line with the perception of suboptimal food acceptance shared by the Portuguese veterinarians.^18^ The cat’s refusal to eat the renal diet was indicated as the main limiting factor, which, based on the remaining findings, could be explained by the apparent inadequate diet introduction by a large proportion of caregivers who did not carry out a gradual transition period or did it for a duration that may have been insufficient. Rather than being the result of poor adherence to veterinary recommendations, whether due to hastiness or misunderstanding, this is probably a consequence of many professionals recommending switching diets immediately or too quickly in this country, as also previously identified.^18^ However, it should be noted that although the guidelines typically recommend a gradual transition of 4 weeks or more,^3^ the most appropriate period depends on each patient and may be shorter in cats that have a good appetite and are more receptive to change, or slower in more selective/‘fussy’ eaters and/or unwell cats. Regarding the latter scenario, most did indeed show clinical signs at diagnosis, particularly weight loss, so their appetite and willingness to accept new foods were probably already compromised. Elliot et al^17^ recently reported that veterinary recommendation was the factor that most frequently influenced UK owners to feed their cat a renal diet. It is expected that the same would have been verified with the Portuguese caregivers, suggesting that if they had allegedly received more effective counselling at the time of the dietary recommendation, namely by informing them of all the potential benefits, their adherence might have been higher. For example, although it depends on several factors such as disease stage and associated comorbidities, previous studies have suggested that a cat on a renal diet may potentially live 2–3 times longer than one fed a non-renal diet,^15,16^ which, if communicated, could make caregivers more prone to keep their cat on a renal diet long term. Yet, only 2.5% of caregivers mentioned being aware of this benefit, revealing an opportunity for improvement for the veterinary team. Therefore, to increase the success rate of its implementation, this therapeutic diet should be initiated as early as clinically recommended, when appetite is not impaired and the cat is apparently stable, and then gradually introduced, according to each cat’s individual response.^3,5^

Most caregivers were administering at least one medication, with antiproteinuric drugs being the most frequently listed, which is aligned with the significant percentage of cats that were proteinuric at diagnosis. Intervention to address proteinuria is advised, as it has been considered a negative prognostic factor.^25,26^ Systemic hypertension has also been associated with the development and aggravation of proteinuria, even though their correlation has not yet been well established and the reverse also seems to occur.^22,27,28^ Therefore, drugs for treating hypertension were also reported among the medications most frequently administered, albeit to a lesser extent. In fact, as CKD progresses, the risk of developing hypertension tends to increase,^29^ which may explain the higher number of cats on treatment for this condition compared with those identified as hypertensive at diagnosis. Even so, this discrepancy may also be the result of more cats actually having hypertension at presentation than were reported (particularly among those whose caregivers were unsure or could not remember) and/or due to the fact that Semintra (Boehringer Ingelheim) was listed as an option for the treatment of both hypertension and proteinuria, which may have caused misleading selection. Another noteworthy finding was that less than 60% of cats diagnosed with these two conditions were reportedly on appropriate medication when the caregiver responded to the questionnaire, suggesting that the remainder were either not receiving the recommended treatment or no longer required it because of disease stabilisation. Future studies should explore in greater depth this potential inconsistency and the criteria being used to support therapeutic decisions regarding these drugs.

Phosphate binders were commonly used by respondents; however, in some cases, their administration may not have aligned with best practice. For instance, over one-third of caregivers reported giving phosphate binders without regard for feeding times, which can reduce their effectiveness.^2,3^ In addition, 17.9% of those using phosphate binders had not attempted a transition to a renal diet, which has been considered the recommended first-line approach for managing hyperphosphataemia.^3,6^

Approximately half of the cats were receiving subcutaneous fluids, a therapy frequently prescribed by veterinarians in this country.^18^ In most of those cases, the procedure was carried out at home, which, according to the findings described by Cooley et al,^13^ was considered an ‘easy’ and ‘no stress’ experience by the majority of the owners surveyed. However, evidence of its long-term benefits, as well as the ideal patient selection criteria, the target fluid volume to be administered and the most suitable treatment regimen, are still lacking.^4,7^ This, combined with the fact that it may potentially be associated with adverse effects, particularly in cats with underlying cardiovascular disease and/or hypoproteinaemia,^7,30^ and that some owners have been reported to administer fluids without assessing their cat’s hydration status,^13^ suggests that this therapy should be carefully considered on a case-by-case basis. Thus, the authors suggest that other strategies to increase water intake, such as feeding wet food and providing several separated water bowls and water fountains, should be prioritised before prescribing in-home fluid therapy, as was already done by many (but not all) of the caregivers surveyed. Regarding ESA therapy, only a minority of the cats were undergoing it, as expected, since this type of treatment has generally been reserved only for symptomatic anaemia or when the packed cell volume is persistently below 20%.^3,11^ Finally, it should be noted that cats were more subjected to medication, nutraceuticals/supplementation, subcutaneous fluids and ESAs when they were diagnosed at late IRIS CKD stages, emphasising the greater frequency of secondary complications at a more advanced disease state.^19,31^ Therefore, less care was required to be provided at home by caregivers with cats diagnosed in early stages, reducing their burden.

Despite all the precautions and procedures normally recommended for managing the disease, a majority of the caregivers considered that the relationship with their cat did not change after diagnosis, which shows that a normal interaction can be preserved without any significant detriment to the existing emotional bond. Interestingly, in approximately one-quarter of the respondents, this connection even seemed to have improved because of greater contact and consequent interaction, albeit it cannot be ruled out that this may have resulted from the cat being more tolerant and cooperative due to unwellness. In contrast, the impact was viewed as negative by a few caregivers because of the forced medication administration, an issue that has been subject to research. Providing caregivers with a demonstration of how to administer the medication in the most effective and cat-friendly way, and choosing products ideally in liquid form, registered for cats and with greater palatability, are some of the recommendations to overcome this difficulty and minimise the associated stress.^32–34^

Although a high percentage of the caregivers surveyed felt fully informed by their veterinarian, 18.3% still had questions, which may have led to some of the misconceptions about the renal diet and the inappropriate in-home management practices reported. When they were unclear, respondents often used the internet for guidance, which could include not only websites with professionally reviewed information but also unsupervised sources with inaccurate advice. Considering the importance of evidence-based veterinary medicine,^35^ a collective effort must be made by the veterinary community to ensure that caregivers are fully informed, reducing the need to seek information through alternative channels. To facilitate this, more veterinary educational resources with easy-to-understand and easy-to-apply content should be developed, filling the gap highlighted by 31.6% of respondents.

One of the main relevant findings of the survey-based study of Portuguese veterinarians was the concerning discrepancy found between the recommended monitoring frequency and the periodicity truly achievable in most cases,^18^ which was further explored here: 70.9% of veterinarians in the preceding study stated they recommended clinical monitoring every 2–3 months or more often in their stable patients, whereas only 50.4% of the caregivers in the present study indicated having received this same instruction. Furthermore, given that the majority of caregivers believed they were adhering to the reassessment periodicity advised, a possible miscommunication between veterinary professionals and their clients was suggested. Still, at least for the late stages, a significantly higher recommended frequency was reported, which is consistent with the current guidelines, since uraemic crises are more frequent and quality of life can deteriorate faster.^2,31^ Apart from raising awareness of the importance of more periodic check-ups, an attempt should be made to minimise the associated stress, as concerns about this were pointed out by non-compliant caregivers. Careful preparation of veterinary visits and cat-friendly handling techniques should be ensured to overcome this limiting factor.^36,37^ Pre-appointment gabapentin is also a commonly used option; however, in these patients, a dose reduction is advisable given the renal impairment.^38,39^

An increased caregiver burden would be expected among this population, as has been described for this and other chronic feline diseases.^40–43^ Nevertheless, only 20% of caregivers clearly stated that their experience would influence their decision to get a new cat in the future, suggesting that for most of them, the challenges and difficulties reported did not outweigh the positive impact of the human–animal bond on their lives.^44,45^ However, as entries relating to already deceased cats were not accepted, it is possible that caregivers who experienced greater distress due to the disease may be underrepresented, namely those who immediately chose euthanasia upon diagnosis. In any case, strategies to ensure continuous caregiver support should be promoted in all cases to minimise this potentially negative impact of the disease.

This study has some limitations that should be acknowledged. Given the absence of a national registry for this feline disease (unlike for cancer^46^), it was not possible to estimate the target population size or characterise it, thereby precluding a proper assessment of the risk of sampling bias. Beyond that, the questionnaire was time-consuming and could only be completed online, which may have led to a greater representation of more dedicated caregivers, while limiting participation to those with internet access. Since respondents had to recall past information, particularly about the diagnosis, some difficulty may have been also encountered, occasionally resulting in more ‘don’t know/don’t remember’ responses or potential loss of data accuracy. Conformity bias cannot be ruled out either, although the assurance of anonymity and the use of neutral wording are expected to have minimised this potential issue. Finally, considering the overrepresentation of female respondents, it is conceivable that the present findings may not adequately reflect the views of male cat caregivers. This is a frequent issue in research, with other recent questionnaire-based studies on cat owners reporting participant pools in which women accounted for more than 90% of those surveyed.^17,47,48^ Greater willingness to respond to online surveys,^49,50^ more use of social media,^51^ and increased bonding with and concern for their cat^52,53^ may explain this female predominance. However, it is also likely that more women than men are taking on the role of primary caregiver for domestic cats, explaining this higher proportion, which may in fact be consistent with the target population.

Despite this, to the best of the authors’ knowledge, this is one of the most comprehensive caregiver survey-based studies on feline CKD published to date, and the first to be conducted in a southern European country. Another distinguishing feature was the decision not to include entries regarding cats that were already deceased at the time of completion, as this loss could have negatively influenced some of the answers and would not reflect current veterinary care. Furthermore, the final response rate was considered satisfactory, especially if compared with other similar survey-based studies with longer collection periods^14^ and/or larger target populations,^12–14,17,54^ adequately representing the population group under investigation.

## Conclusions

This study showed that most caregivers in Portugal felt supported by their veterinarian in managing feline CKD, although several inadequate practices were being carried out, such as lack of BP measurement at diagnosis, potentially improper transition to a renal diet and incorrect administration of phosphate binders. The importance of early diagnosis was also highlighted, as it was associated with a lower prevalence of clinical signs and less in-home care. Overall, study findings showed that veterinarian–caregiver communication should be refined to ensure better adherence, maximising therapeutic success.

## Supplemental Material

Online questionnaire provided to Portuguese caregivers of cats diagnosed with chronic kidney disease ’Feline chronic kidney disease from the caregivers’ perspective’.
