# Supplementary material for: Caregivers’ perspectives on feline chronic kidney disease in Portugal: a questionnaire-based study
Source: J Feline Med Surg. 2025 Dec 15;27(12):1098612X251377486. doi: 10.1177/1098612X251377486 (PMC12709014; doi:10.1177/1098612X251377486)
Supplement: Online questionnaire provided to Portuguese caregivers of cats diagnosed with chronic kidney disease [file sj-docx-1-jfm-10.1177_1098612X251377486.docx]

**Supplemental material**

Online questionnaire provided to Portuguese caregivers of cats diagnosed with chronic kidney disease: "Feline chronic kidney disease from the caregivers’ perspective" (original questionnaire language: Portuguese)

**Feline chronic kidney disease from the caregivers' perspective**

This questionnaire aims to collect information about cats diagnosed with chronic kidney disease from the caregivers’ perspective. Thus, it is intended for all people over 18 years old, who live in Portugal (mainland and autonomous regions) and who are currently caring for a cat with this disease. If you have more than one cat with this condition, please complete the questionnaire for each cat individually.

In terms of structure, the questionnaire is composed of several closed-ended and short-answer questions, and an optional open-ended question at the end, which will take no more than 15 to 20 minutes in total to complete. The questions you will be asked are related to your cat’s characteristics, habits and environment, the diagnosis and treatment implemented, and also your own perception on this topic.

Completing the questionnaire is anonymous and voluntary, and no personal information that potentially identifies you will be required or documented. All data collected from your responses will be treated with the utmost confidentiality and used solely for scientific purposes. For any additional questions regarding the privacy policy applied to this study, we suggest that you consult the privacy statement of the University of Trás-os-Montes and Alto Douro (<https://www.utad.pt/app-privacy-policy>).

While completing the questionnaire, if you have any questions or comments to make, please contact the principal investigator (Tomás Magalhães) using the following e-mail tomas.rodrigues.magalhaes@gmail.com.

Thank you in advance for your collaboration and the time you will spend answering our questions.

**Declaration of participation**

1. Do you voluntarily agree to participate in this study and authorize the use of your answers for scientific purposes?
   - Yes
   - No

**Caregiver’s information**

1. With which gender do you identify? (select only one option)
   - Male
   - Female
   - Non-binary
   - Prefer not to say
2. Which age group are you in? (select only one option)
   - 18 to 24 years old
   - 25 to 34 years old
   - 35 to 44 years old
   - 45 to 54 years old
   - 55 to 64 years old
   - More than 65 years old
3. What is the highest level of education you completed? (select only one option)
   - Basic education
   - Upper secondary education
   - Bachelor’s degree
   - Master’s degree
   - Doctoral degree (PhD)
4. In which district/ region of the country do you live with your cat? (select only one option)
   - Aveiro
   - Beja
   - Braga
   - Bragança
   - Castelo Branco
   - Coimbra
   - Évora
   - Faro
   - Guarda
   - Leiria
   - Lisboa
   - Portalegre
   - Porto
   - Santarém
   - Setúbal
   - Viana do Castelo
   - Vila Real
   - Viseu
   - Santa Maria Island
   - São Miguel Island
   - Terceira Island
   - São Jorge Island
   - Pico Island
   - Graciosa Island
   - Faial Island
   - Flores Island
   - Corvo Island
   - Madeira Island
   - Porto Santo Island
5. Have you had experience caring for a cat with chronic kidney disease in the past? (select only one option)
   - Yes
   - No

**Cat characteristics, habits, and environment**

1. How old, in years, was your cat when he/she was diagnosed with chronic kidney disease? ____.
2. What is your cat’s breed? (select only one option)
   - Bengal
   - British shorthair
   - British longhair
   - Domestic shorthair
   - Domestic longhair
   - European
   - Exotic
   - Norwegian forest cat
   - Undefined breed cat
   - Maine Coon
   - Persian cat
   - Siamese
   - Sphynx
   - Other: ___.
3. What was your cat’s reproductive status at the time of diagnosis? (select only one option)
   - Intact
   - Neutered
   - I don’t know
4. In addition to chronic kidney disease, has your cat been diagnosed with any other disease? (select one or more options)
   - Diabetes mellitus
   - Cardiovascular disease
   - Inflammatory bowel disease (IBD)
   - Osteoarthritis (problems in bones and joints)
   - Periodontal disease (problems in the oral cavity, such as gingivitis/ periodontitis)
   - Polycystic kidney disease
   - Cancer
   - Hyperthyroidism
   - Feline Immunodeficiency Virus (FIV)
   - Feline Leukemia Virus (FeLV)
   - Urolithiasis (urinary stones)
   - Liver disease
   - Pancreatic disease
   - No
   - I don't know/ I don't remember
   - Other: ____.
5. Regarding the environment where your cat lives, which of the following conditions apply? (select one or more options)
   - Access to automatic water fountains with continuous water flow
   - Number of litter boxes higher than total number of cats
   - Distribution of litter boxes in different rooms of the house (if you have more than one litter box)
   - Several water bowls distributed in different spaces of the house
   - Environment shared with other cats
   - Water bowls placed next to food bowls
6. What type of diet did you give your cat before the diagnosis of chronic kidney disease? (select one or more options)
   - Dry commercial food
   - Wet commercial food (such as pâté, mousse, or chunks in jelly)
   - Homemade food
   - Raw food
7. In terms of composition, the food you previously provided was intended to cover the nutritional needs of which group of cats? (select only one option)
   - Kittens *(branching to question 16)*
   - Adult cats *(branching to question 16)*
   - Senior cats *(branching to question 16)*
   - Cats with health problems/ food intolerances (therapeutic diets) *(branching to question 15)*
   - I don’t know *(branching to question 14)*
8. Please indicate the brand/ commercial name of the food: ____.
9. For the nutritional management of which disease was the previous therapeutic diet intended? (select one or more options)
   - Food allergy
   - Diabetes mellitus
   - Gastrointestinal disorders
   - Obesity
   - Dermatological/ skin conditions
   - Other: ____.

**Aspects related to the clinical presentation and diagnosis**

1. What signs did your cat show at the time of diagnosis? (select one or more options)
   - Weight loss
   - Loss of appetite/ “fussy” appetite
   - Vomiting
   - Increased water intake (your cat was drinking more than usual)
   - Increased urine production (your cat was urinating more than usual)
   - Lethargy/ weakness (your cat was more tired than usual)
   - No clinical signs. He/ she was diagnosed through routine tests.
   - Other: ____.
2. What tests did your veterinarian perform on your cat to establish the diagnosis? (select one or more options)
   - Blood tests
   - Urine analysis
   - Abdominal ultrasound
   - I don't know/ I don't remember
   - Other: ____.
3. Was the presence of protein in the urine (proteinuria) assessed in your cat when he/she was diagnosed? (select only one option)
   - Yes, and the measured parameter (urinary protein-to-creatinine ratio) was normal
   - Yes, and the measured parameter (urinary protein-to-creatinine ratio) was abnormal
   - Yes, but I don't know/ I don't remember the result
   - No
   - I don't know/ I don't remember
4. Was your cat's blood pressure measured when he/she was diagnosed? (select only one option)
   - Yes, and it was normal
   - Yes, and it was high (hypertension)
   - Yes, and it was low (hypotension)
   - Yes, but I don't know/ I don't remember the result
   - No
   - I don't know/ I don't remember
5. Did your veterinarian explain the staging system for feline chronic kidney disease and/or indicate your cat's disease stage at the time of diagnosis? (select only one option)
   - Yes *(branching to question 21)*
   - No *(branching to question 22)*
   - I don't know/ I don't remember *(branching to question 22)*
6. What was the stage of your cat's disease at the time of diagnosis? (select only one option)
   - 1
   - 2
   - 3
   - 4
   - I don't know/ I don't remember

**Details about nutritional management**

1. After the diagnosis of chronic kidney disease, did you receive a recommendation to start a therapeutic kidney diet for your cat? (select only one option)
   - Yes *(branching to question 23)*
   - No *(branching to question 24)*
2. Who gave you that nutritional recommendation? (select only one option)
   - Veterinarian
   - Veterinary nurse
   - Veterinary assistant and/or receptionist
   - Pet shop employee or breeder
   - Other: ____.
3. Are you aware of the benefits of the kidney diet for controlling your cat's disease? (select only one option)
   - Yes *(branching to question 25)*
   - No *(branching to question 26)*
4. Specify which benefits your veterinarian told you about: ____.
5. Have you transitioned your cat to a therapeutic kidney diet? (select only one option)
   - Yes (*branching to question 27)*
   - No (*branching to question 31)*
6. How long did it take to complete the transition from the previous diet to the kidney diet? (select only one option)
   - I did not transition the diet / I immediately changed the diet
   - Less than 1 week
   - 1 to 2 weeks
   - 3 to 4 weeks
   - 5 to 6 weeks
   - 7 to 8 weeks
   - More than 8 weeks
7. What approximate percentage of your cat's daily food intake does the kidney diet currently represent? (select only one option)
   - 100% (kidney diet represents all of the daily food intake) (*branching to question 30)*
   - Less than 100%, but more than 75% of the daily food intake (*branching to question 29)*
   - Less than 75%, but more than 50% of the daily food intake (*branching to question 29)*
   - Less than 50%, but more than 25% of the daily food intake (*branching to question 29)*
   - Less than 25% of the daily food intake (*branching to question 29)*
   - 0% (kidney diet is not part of my cat’s daily food intake) (*branching to question 29)*
8. Why does the kidney diet not represent the entirety of your cat’s daily food? (select one or more options)
   - My cat does not like this type of diet, rejecting it partially or completely
   - I do/ did not notice any improvement in my cat's general condition
   - The cost of the kidney diet is too high
   - Lack of availability and/or access to this type of diet
   - Other: ____.
9. What type(s) of kidney food do you include in your cat's daily diet? (select one or more options)
   - Dry commercial food
   - Wet commercial food (such as pâté, mousse or chunks in jelly)
   - Homemade food
   - Raw food
   - Not applicable

**Treatment approaches**

1. Which of the following medications do you administer to your cat? (select one or more options)

Note: Select “Other” only if you do not know or if you do not consider that the medication you are administering to your cat fits any of the other options and please indicate the name written on the package.

- - Antiemetic (for nausea and vomiting). Example: Cerenia^®^.
  - Appetite stimulant. Example: Oral mirtazapine or mirtazapine for cutaneous application on the inner pinna of the cat’s ear (Mirataz^®^).
  - Medication to control hypertension. Examples: Amodip^®^ or Semintra^®^.
  - Medication to control proteinuria (excessive protein in the urine). Examples: Banacep^®^, Fortekor^®^, Benakor^®^, or Semintra^®^.
  - Proton pump inhibitors for gastric protection. Example: Omeprazol.
  - Histamine Type-2 Receptor Antagonists for gastric protection. Example: Lasa^®^.
  - None
  - Other: ____.

1. Which of the following supplements do you administer to your cat? (select one or more options)

Note: Select “Other” only if you do not know or if you do not consider that the supplement you are administering to your cat fits any of the other options and please indicate the name written on the package.

- - Phosphate binders (to reduce blood phosphate levels). Examples: WeNefro^®^, Renal P^®^, or Ipakitine^®^.
  - Omega-3 fatty acids. Examples: Coatex^®^, KimiDerm^®^, Megaderm^®^, Omnicutis^®^, Omniomega^®^, Redonyl^®^, or WeDerm^®^.
  - Iron and B vitamins. Examples: WeHemo^®^ or Red Cell Care^®^.
  - Potassium. Example: Kaminox^®^.
  - Multivitamin concentrates. Examples: GimCat Multi-Vitamin^®^, Purina^®^ Pro Plan^®^ Multivitamins+, or Urano Vet U-Vita^®^.
  - None
  - Other: ____.

1. If you administer a phosphate binder, are you doing it in combination with the kidney diet? (select only one option)
   - Yes
   - No
   - Not applicable
2. Regardless of the type of diet, how do you administer the phosphate binder? (select only one option)
   - Mixed with food in a single meal
   - Mixed with food in two or more meals
   - Directly in the mouth, regardless of mealtimes
   - Not applicable
   - Other: ____.
3. Does your cat receive any type of injection given by your veterinarian to treat anaemia (decrease in red blood cells) secondary to chronic kidney disease? (select only one option)
   - Yes
   - No
   - I don't know/ I don't remember
4. Does your cat receive subcutaneous fluids (under the skin) to control dehydration? (select only one option)
   - Yes, in the clinic (*branching to question 38)*
   - Yes, at home (*branching to question 38)*
   - Yes, in the clinic and at home (*branching to question 38)*
   - No (*branching to question 37)*
   - I don't know/ I don't remember (*branching to question 38)*
5. Why is subcutaneous fluid therapy not part of your cat's treatment? (select one or more options)
   - It was not recommended by my veterinarian
   - My cat does not tolerate the procedure
   - I do not want to subject my cat to this type of procedure
   - I don’t/ didn't notice any improvement in my cat's general condition
   - Due to the cost associated with fluid therapy sessions
   - Lack of time
   - Other: ____.

**Relationship with your cat and with your veterinarian**

1. How did the diagnosis of chronic kidney disease affect the emotional bond you have with your cat? (select only one option)
   - It got worse (*branching to question 39)*
   - It is the same (*branching to question 41)*
   - It got better (*branching to question 40)*
   - I don't know (*branching to question 41)*
2. What are the main reasons for this negative effect on the emotional bond with your cat? (select one or more options)
   - We are in a conflict created due to the forced administration of drugs and/or supplements
   - My cat has developed avoidance behaviour towards me because I'm the one who takes him/her to the clinic for tests and treatments
   - Loss of bonding, because my cat has reduced interaction with me (less time available for cuddles and play)
   - Due to the distance I created in an attempt to respect his/her space since he/she is now ill
   - Other: ____.
3. What are the main reasons for this positive effect on the emotional bond with your cat? (select one or more options)
   - Greater contact because of the new routines, such as the drug/ supplement administration and the visits to the veterinary clinic/ hospital
   - Greater approximation due to my constant concern about my cat's general condition
   - Increased interaction due to positive change in my cat's behaviour/ temperament
   - Other: ____.
4. Do you feel informed by your veterinarian regarding the diagnosis, treatment, and monitoring of your cat's disease? (select only one option)
   - Yes, completely. All my questions were answered. (*branching to question 43)*
   - Yes, partially. Some questions were left unanswered. (*branching to question 42)*
   - No (*branching to question 42)*
5. Which sources did you use to answer your questions? (select one or more options)
   - Second opinion with another veterinarian
   - Veterinary nurse at the same or another veterinary clinic/ hospital
   - Didactic material available at my veterinary clinic/ hospital (examples: flyers, brochures, and posters)
   - Internet (examples: social media, forums and blogs)
   - Books or magazines
   - Family or friends
   - Pet shop employees or breeders
   - None. I did not look for more information to answer my questions.
6. Considering your cat in a stable phase of the disease, how often does your veterinarian recommend that you schedule a check-up for a general examination and blood and urine tests? (select only one option)
   - More than once a month
   - Monthly
   - Every 2 to 3 months
   - Every 4 to 5 months
   - Every 6 months
   - Every 7 or more months
   - Only when my cat gets worse
7. Do you generally comply with this monitoring frequency recommended by your veterinarian? (select only one option)
   - Yes (*branching d to question 46)*
   - No (*branching to question 45)*
8. What are the reasons for not following the medical recommendation? (select one or more options)
   - I do not think my cat needs to be monitored so often
   - Reassessment appointments and tests are too expensive for me to guarantee the recommended frequency
   - I do not have time to take my cat to the veterinary clinic/ hospital so often
   - My cat becomes very stressed during transportation and/or at the veterinary clinic/ hospital
   - I live far from the veterinary clinic/ hospital
   - I forget to schedule an appointment
   - Other: ____.
9. Finally, considering the statements presented below, which do you think are true for you? (select one or more options)
   - Chronic kidney disease is curable with proper treatment and monitoring
   - Chronic kidney disease has no cure, and treatment only aims to slow its progression and improve my cat's quality of life
   - The available information about this disease is sufficient to understand its general aspects
   - More educational resources are needed to better understand this disease
   - The impact that this disease has on my cat and on my life will influence future decisions regarding the purchase/ adoption of a new cat
   - The impact that this disease has on my cat and on my life will not influence future decisions regarding the purchase/ adoption of a new cat

**Final comments (optional)**

1. If you would like to leave a comment about your own experience as a caregiver of a cat with chronic kidney disease, please feel free to use the space below.

**Thank you for your collaboration!**
